# Experimental verification of the rotational type of chiral spin spiral structures by spin-polarized scanning tunneling microscopy

**DOI:** 10.1038/s41598-017-13329-9

**Published:** 2017-10-16

**Authors:** Masahiro Haze, Yasuo Yoshida, Yukio Hasegawa

**Affiliations:** 1 Institute for Solid State Physics, University of Tokyo, Kashiwa, Chiba 277-8581, Japan; 2 Present Address: Department of Physics, Kyoto University, Kyoto 606-8502, Japan

## Abstract

We report on experimental verification of the rotational type of chiral spin spirals in Mn thin films on a W(110) substrate using spin-polarized scanning tunneling microscopy (SP-STM) with a double-axis superconducting vector magnet. From SP-STM images using Fe-coated W tips magnetized to the out-of-plane and [001] directions, we found that both Mn mono- and double-layers exhibit cycloidal rotation whose spins rotate in the planes normal to the propagating directions. Our results agree with the theoretical prediction based on the symmetry of the system, supporting that the magnetic structures are driven by the interfacial Dzyaloshinskii-Moriya interaction.

## Introduction

In magnetic systems, competition between exchange interactions and magnetic anisotropy determines the ground state. In a case where the inversion symmetry of the system is broken, the antisymmetric exchange interaction called the Dzyaloshinskii-Moriya interaction (DMI)^1,2^ plays a significant role and causes formation of complex chiral spin structures, such as skyrmion lattices^3–5^, domain walls^6,7^, and homogeneous spin spiral structures^8–10^. Since the inversion symmetry is naturally broken at interfaces, 3 *d* magnetic ultrathin films formed on 5 *d* non-magnetic heavy-elemental substrates often exhibit non-collinear chiral spin structures driven by the interfacial DMI (iDMI)^11–18^. In magnetic structures driven by iDMI, it is predicted that spins are rotating in the plane parallel to the propagating axis, called Néel or cycloidal rotations, with unique rotational senses (right- or left-handed). Therefore, to verify whether iDMI plays a dominant role for the system, it is important to characterize explicit spin structures of the system including rotational type and sense down to the atomic scale.

Spin-polarized scanning tunneling microscopy (SP-STM) has been utilized for this purpose with multi-axis superconducting vector magnets^12,13,18^. Meckler *et al*. performed SP-STM measurements on Fe double layer (DL) on W(110) with a triple-axis vector magnet, determining that rotational type of the domain wall is Néel-type^13^. Heinze *et al*. revealed the Néel-type skyrmion magnetic ground state of Fe monolayer (ML) on Ir(111) by SP-STM measurements with a double-axis vector magnet^12^. In our previous work, we conducted an SP-STM measurement on Mn ML and DL formed on W(110) with a double-axis vector magnet, and clarified the unique rotational senses of homogeneous spin-spirals^18^, indicating that the magnetic structures are driven by iDMI. The rotational types are, however, still undefined experimentally whereas the cycloidal type of spin spirals are theoretically predicted as ground states^16,19^.

In this study, we further investigate Mn ML and DL /W(110) with SP-STM with a double-axis vector magnet, and provide experimental proofs that both layers indeed exhibit cycloidal spin spiral rotations. This result agrees well with the previous theoretical predictions from symmetry of the systems^16,19^, which supports that the magnetic structures are driven by iDMI.

## Results

**Figure 1:**
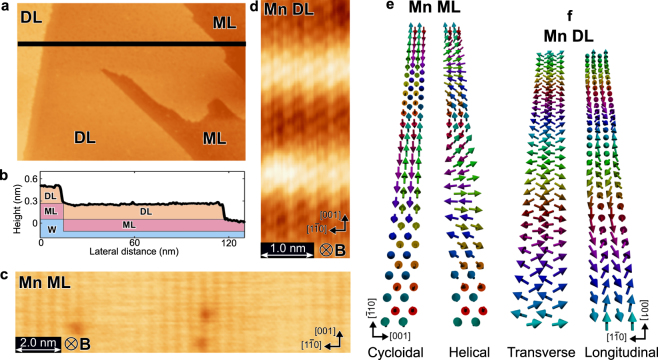
STM images with non-magnetic and magnetic tips of Mn mono- and double-layers on a W(110) substrate and their possible spin structures. (**a**) An typical topographic image of Mn thin films formed on W(110). ML and DL represent regions of monolayer and double layer. (**b**) Cross-sectional profile taken along the solid line in (**a**). (**c**) An SP-STM image taken on the region of Mn ML with an Fe-coated tip. Tunneling current *I*_T_ = 10 nA, sample bias voltage *V*_S_ = 10 mV and external magnetic field *B*_⊥_ = 1 T. (**d**) A spin-polarized *dI*/*dV* image taken on Mn DL. *I*_T_ = 50 nA, *V*_S_ = −50 mV and *B*_⊥_ = 2 T. (**e**) Schematics of two possible spin structures of Mn ML, called cycloidal and helical rotations, respectively. In a cycloidal rotation, spin spiral propagates along the [1$\bar{{\rm{1}}}$0] direction with the spins rotating in the (001) plane. In a helical rotation, spin spiral propagates along the [1$\bar{{\rm{1}}}$0] direction with the spins rotating in the (1$\bar{{\rm{1}}}$0) plane. (**f**) Schematics of two possible spin structures of Mn DL. In a transverse conical rotation, spin spiral propagates along the [001] direction with rotating in the (1$\bar{{\rm{1}}}$0) plane. In a longitudinal rotation, spin spiral propagates along the [001] direction with rotating in the (001) plane.

Figure 1a and b show a typical topographic image and a corresponding line profile on Mn ML and DL. Figure 1c shows an SP-STM image taken on an ML region of Mn/W(110) with a tip magnetized perpendicular to the sample surface with *B*_⊥_ = 1 T. Since an SP-STM signal depends on the cosine of the angle *θ* between the tip and sample magnetization directions, bright and dark rows separated by an atomic distance along the [1$\bar{{\rm{1}}}$0] direction (0.47 nm) indicate that the Mn rows have parallel and antiparallel magnetization components with respect to the tip magnetization direction. The contrast vanishes at the Mn rows whose magnetization directions are close to the in-plane direction (i.e., *θ* is close to 90°). Figure 1e shows schematics of two possible spin spiral structures that can reproduce the SP-STM image; cycloidal and helical spin spirals^16,18^. Although we experimentally confirmed that the rotational sense is left-handed as mentioned before^18^, there is still an experimental ambiguity about the rotational type of the spin spiral, that is, helical or cycloidal spin structure.

The situation of Mn DL is similar to the case of Mn ML. Figure 1d shows an atomically-resolved spin-polarized *dI*/*dV* image taken on a DL region of Mn/W(110). Bright and dark rows separated by an atomic distance along the [1$\bar{{\rm{1}}}$0] direction indicate that the Mn rows in DL also have parallel and antiparallel components. In addition, a sinusoidal pattern with 2 nm periodicity along the [001] direction is observed, indicating a spin-spiral structure propagating to the [001] direction. In this case, we can think of two possible spin structures as shown in Fig. 1f, transverse^19^ (the left panel) and longitudinal (the right panel) conical spin-spiral structures with antiferromagnetic coupling along the [1$\bar{{\rm{1}}}$0] direction, but the experimental confirmation has not been done yet. We note that spins rotate in the plane normal (parallel) to the propagating axis in cycloidal and transverse conical (helical and longitudinal conical) spin spirals.

**Figure 2:**
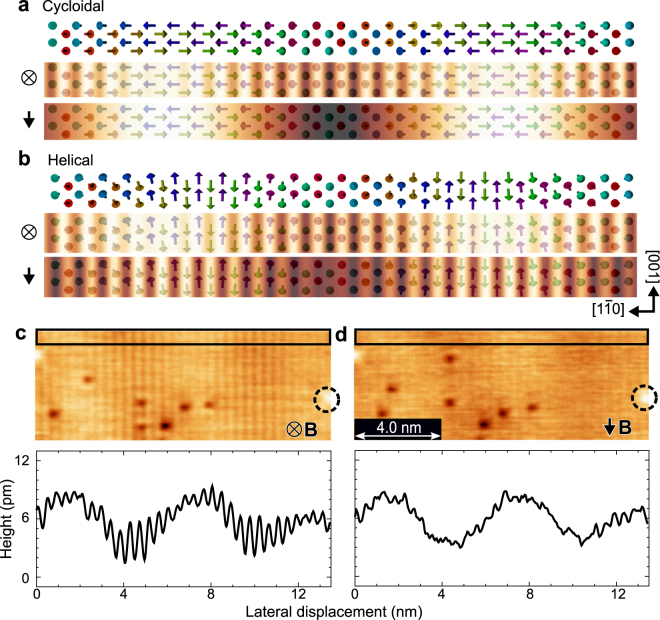
Simulated SP-STM images for Mn monolayer and experimental confirmation of a cycloidal spin spiral. (**a,b**) Schematic spin structures of a cycloidal and helical type rotation of Mn ML, respectively. Middle and lower panels represent simulated spin-polarized and TAMR signals with tips magnetized to the out-of-plane and [001] directions, respectively. (**c,d**) SP-STM images and the corresponding cross-sectional profiles averaged in the boxed areas in the images. Both are taken with tips magnetized to the out-of-plane and the [001] directions, respectively. *I*_*T*_ = 40 nA, *V*_*S*_ = 15 mV. *B*_⊥_ = 2 T for (**c**) and ${B}_{\parallel }$ = 1 T for (**d**).

First we focus on SP-STM measurements on Mn ML with a tip magnetized along the [001] direction. If the rotational type is cycloidal, the magnetic contrast will be considerably suppressed as shown in Fig. 2a while it remains with 90 ° phase shift from the out-of-plane tip image for the case of a helical spin spiral. Therefore, this measurement provides a conclusive evidence for the type of spin spiral structure. Note that, a sinusoidal pattern along the [1$\bar{{\rm{1}}}$0] direction with 6 nm periodicity, called tunneling anisotropic magnetoresistance (TAMR)^20,21^, always appears, which is maximized (minimized) in the area where the sample magnetization is the in-plane (out-of-plane) direction. This corrugation arises from electronic states induced by the spin orbit interaction and can be detected even with a non-magnetized tip.

The upper panels of Fig. 2c and d show SP-STM images obtained with tips sensitive to the out-of-plane component of the sample magnetization and the component along the [001] direction, respectively. We obtained the two images in the same field of view, as evidenced by a white-dot marker (circled with a dashed line) that appears on the right edge of the images. Some of the impurities (i.e., dark spots) changed their positions during the imaging. Both the magnetic and TAMR components are visible with the out-of-plane tip (Fig. 2c). In contrast, in the SP-STM image taken with the [001]-magnetized tip (Fig. 2b), the magnetic contrast is strongly suppressed while the TAMR signal stays the same. We can demonstrate the suppression of the magnetic signal more clearly in the averaged cross-sectional profiles (the lower panels) collected in the boxed areas of Fig. 2c and d. A faint magnetic contrast found in Fig. 2d is presumably due to small misalignment of the tip magnetization direction. Based on these observations, we conclude that Mn ML has a cycloidal spin spiral structure, in good agreement with the theoretical prediction based on first principles calculations^16^.

**Figure 3:**
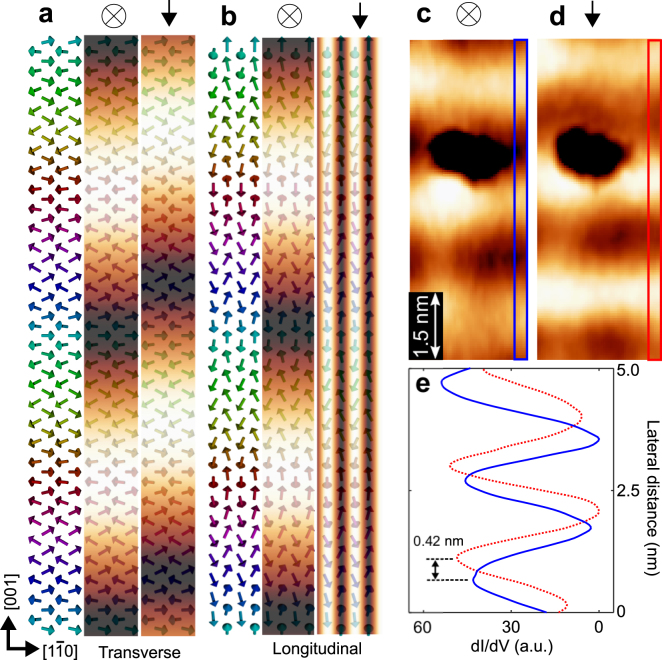
Simulated SP-STM images for Mn double-layer and experimental confirmation of a transverse conical spin spiral. (**a,b**) Schematic spin structures of a cycloidal and helical type rotation of Mn DL, respectively. Middle and right panels represent simulated spin-polarized signals with tips magnetized parallel to the out-of-plane and [001] directions, respectively. **(c,d)** Spin polarized *dI*/*dV* images taken with tips magnetized parallel to the out-of-plane and [001] directions, respectively. *I*_*T*_ = 1 nA, *V*_*S*_ = −50 mV. *B*_⊥_ = 2 T for **(c)** and ${B}_{\parallel }$ = 1 T for **(d)**. **(e)** Blue solid and red dotted line indicate the cross-sectional profiles taken in the boxed areas in **(c)** and **(d)**, respectively.

Next, we determined the rotational type of Mn DL in the same way as in the case of Mn ML. Fig. 3a and b show schematic spin structures of the transverse and longitudinal conical structures with simulated SP-STM signals. The transverse and longitudinal conicals have cycloidal and helical type spin rotations with respective to the propagation axis, respectively. For both rotational types, SP-STM signals are a sinusoidal pattern with 2 nm periodicity along the [001] direction with an out-of-plane magnetized tip. By using a tip magnetized to the [001] direction, this sinusoidal pattern shifts the phase by 90° for the transverse conical while the sinusoidal pattern vanishes and a short periodic pattern with the atomic distance along the [1$\bar{{\rm{1}}}$0] direction shows up for the longitudinal conical, reflecting the antiferromagnetic coupling along the [1$\bar{{\rm{1}}}$0] direction.

Figure 3c and d show experimental spin-polarized *dI*/*dV* images of Mn DL/W(110) taken on the same area with tips magnetized along the out-of-plane and [001] directions, respectively. A sinusoidal pattern along the [001] direction shifts the phase between the images, which is more clearly observed by taking line profiles along the patterns of the images (Fig. 3e). The sinusoidal pattern shifts by 0.42 nm, which roughly corresponds to a quarter of the periodicity. This result clearly indicates that the rotational type of Mn DL is transverse conical.

## Discussion

At the interface, inversion symmetry along the out-of-plane direction is broken while the systems have a mirror symmetry with respect to a plane that is parallel to the propagating axis and normal to the interface, i.e., (001) plane for Mn ML and (1$\bar{{\rm{1}}}$0) plane for Mn DL. In these cases, the direction of the DMI vector, if exist, should be normal to the mirror plane, which produces chiral structures whose spins rotate in the mirror plane, e.g., cycloidal spin spiral or Néel type domain walls^2,16^. Our results undoubtedly confirm experimentally that the rotational types of ML and DL are the cycloidal and transverse conical spin spiral structures, respectively, which is consistent with the model of the iDMI-driven spin spiral structures.

## Conclusion

We determined the rotational type of the spin spiral structure observed on Mn ML and DL/W(110) experimentally using SP-STM with an Fe-coated magnetic tip in magnetic fields parallel and perpendicular to the sample surface. With the tip magnetized along the (001) plane, we found strong suppression of the magnetic contrast in Mn ML, indicating that the spin structure of Mn ML is cycloidal spin spiral rotating in the (001) plane. We also revealed the cycloidal rotation of Mn DL in the same fashion. These results are consistent with the prediction from symmetry of the systems, supporting that the magnetic structures are driven by iDMI. This work demonstrates that SP-STM measurements with a double-axis superconducting magnet enable us to explicitly determine complicated surface spin structures down to the atomic scale.

## Methods

The experiments have been performed in low-temperature ultrahigh vacuum (UHV) STM (Unisoku USM-1300S with RHK R9 controller), in which the sample and the tip are cooled down to 5 K. A two-axis superconducting magnet is equipped to apply magnetic fields perpendicular (|*B*_⊥_| ≤ 2 T) and parallel ($|{B}_{\parallel }|\le$ 1 T) to the sample surface. A W(110) substrate is prepared by several cycles of flashing above 2300 K in UHV and annealing at 1500 K in an oxygen atmosphere of 1 × 10^−4^ Pa^22^. We deposit Mn onto the W(110) substrate for 25 s at a deposition rate of 1.5 ML/min from a Ta crucible heated by electron bombardment. In order to avoid the nucleation of additional layers and to achieve step-flow growth of Mn ML, Mn is deposited just after the flashing to ensure high mobility of the deposited atoms^23^. For the preparation of spin-polarized tips, we deposit Fe by electron bombardment heating on an electrochemically etched W tip that had been flashed in UHV to remove surface oxide layers. The Fe-coated W tips usually exhibit in-plane magnetization at zero magnetic field, and the magnetization can be flipped toward the direction of the external magnetic field with |*B*_⊥_| = 2 T or $|{B}_{\parallel }|$ = 1 T^24^. All STM and SP-STM measurements are performed in constant current mode.

### Data availability

The datasets generated during the current study are available from the corresponding author on reasonable request.
